# Turbulent chimeras in large semiconductor laser arrays

**DOI:** 10.1038/srep42116

**Published:** 2017-02-06

**Authors:** J. Shena, J. Hizanidis, V. Kovanis, G. P. Tsironis

**Affiliations:** 1 Crete Center for Quantum Complexity and Nanotechnology, Department of Physics, University of Crete, 71003 Heraklion, Greece; 2 Department of Physics, School of Science and Technology, Nazarbayev University, 53 Kabanbay Batyr Ave, Astana, Republic of Kazakhstan; 3 Institute of Electronic Structure and Laser, Foundation for Research and Technology–Hellas, P.O. Box 1527, 71110 Heraklion, Greece; 4 National University of Science and Technology MISiS, Leninsky prosp. 4, Moscow, 119049, Russia

## Abstract

Semiconductor laser arrays have been investigated experimentally and theoretically from the viewpoint of temporal and spatial coherence for the past forty years. In this work, we are focusing on a rather novel complex collective behavior, namely chimera states, where synchronized clusters of emitters coexist with unsynchronized ones. For the first time, we find such states exist in large diode arrays based on quantum well gain media with nearest-neighbor interactions. The crucial parameters are the evanescent coupling strength and the relative optical frequency detuning between the emitters of the array. By employing a recently proposed figure of merit for classifying chimera states, we provide quantitative and qualitative evidence for the observed dynamics. The corresponding chimeras are identified as *turbulent* according to the irregular temporal behavior of the classification measure.

Semiconductor lasers are enabling components in multiple platform applications spanning optical communication networks to laser surgery and sensing. Recent works include impressive advances in high-speed lasers with low-power consumption, high-power vertical external cavity surface emitting lasers and high-speed beam steering with phased vertical cavity laser arrays. Significant advances have been made in nitride based lasers, record-high temperature operation quantum dot lasers, and the field of nanolasers with ultralow volume and threshold is coming to technological maturity^1^.

Of special importance for next generation applications such as laser radars, is the design of photonically integrated semiconductor laser arrays that consist of a very large number of properly coupled photonic emitters^2^. It is well known that phase locking of an array of diode lasers is a highly effective method in beam shaping because it increases the output power and reduces the overall needed lasing threshold. Recent work on phase-locked laser arrays through global antenna mutual coupling has employed custom made nano-lasers^3^. Moreover, reconfigurable semiconductor laser networks based on diffractive coupling using Talbot geometry have been studied on commercially available vertical cavity diode lasers^4^.

In the present work, we are interested in the collective behavior of a large array of semiconductor lasers with nearest-neighbor interactions. The crucial parameters for the observed dynamics are the coupling strength and the relative optical frequency detuning between the lasers, which introduces realistic inhomogeneities into the system. Our focus, in particular, is to identify the parameter regions where chimera states emerge and subsequently characterize these states using suitable classification measures^5^.

Chimera states were first reported for identical and symmetrically coupled phase oscillators^6^. For over a decade now, a number of works has been dedicated to this phenomenon of coexisting synchronous and asynchronous oscillatory behavior (see ref. 7 and references within). The latest developments in this field involve their study in physical, higher-dimensional systems beyond phase oscillators, their experimental verification^8^,^9^,^10^,^11^,^12^,^13^,^14^,^15^,^16^, their robustness against system inhomogeneities^17^,^18^,^19^,^20^, their existence in stochastic systems^21^, and their manipulation through control techniques^22^,^23^,^24^,^25^.

Coupled lasers have been extensively studied in terms of nonlinear dynamics^26^,^27^,^28^,^29^,^30^ and synchronization phenomena^31^,^32^,^33^,^34^, but works on chimera states in laser networks have appeared only recently. In refs 35 and 36 chimera states were reported both theoretically and experimentally in a virtual space-time representation of a single laser system subject to long delayed feedback. Furthermore, so-called “small chimeras” were numerically observed in a network of four globally delay-coupled lasers in refs 37 and 38, for both small and large delays. Such chimeras exist for very small network sizes and do not require nonlocal coupling in order to emerge. In our study we use neither nonlocal, nor global coupling but simple nearest-neighbor interactions which is physically plausible for lasers, e. g., grown on a single chip. This coupling realization is less expensive computationally. Moreover, it revises the general belief that nonlocal coupling is essential for the existence of chimeras^39^.

We will show that the crucial parameter for the collective behavior in our system is the frequency detuning between the coupled lasers. The effect of detuning has been examined before in ref. 40 but with respect to in- and anti-phase synchronization. Moreover, transitions from complete to partial synchronization (optical turbulence) were explained, for a small array of three lasers. Here, we address the emergence of the hybrid phenomenon of chimera states in a *large* laser array and provide a quantification of these patterns using newly developed classification measures^5^.

## Results

Our system consists of an array of *M* locally coupled semiconductor lasers. The evolution of the slowly varying complex amplitudes 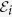 of the electric fields and the corresponding population inversions *N*_*i*_ is given by^41^,^42^:


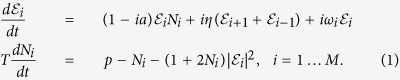


The amplitude-phase coupling is modeled by the linewidth enhancement factor *a* = 5, *T* = 400 is the ratio of the carrier to the photon lifetime of the photons in the laser cavity. The normalized angular frequency *ω*_*i*_ measures the optical frequency detuning of laser *i* from a common reference. The diode lasers are pumped electrically with the excess pump rate *p* = 0.5. These parameters represent typical experimental values from multiple experiments performed in the past 20 years using quantum well laser media^43^. The coupling strength *η* is a control parameter used to tune the dynamics of the system. We have used open boundary conditions to account for the termination of the array in a finite system. By using polar coordinates 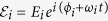 and separating real from imaginary part, we get:


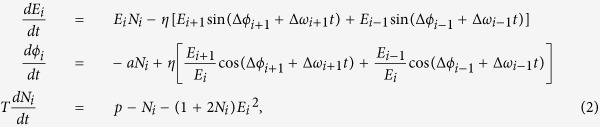


where 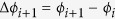, 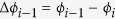, 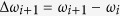, 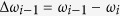. For the special case of *two lasers* and in the absence of detuning, Eq. (2) have the following fixed points:


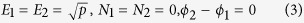



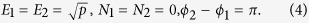


To investigate the stability of these steady states we introduce small perturbations and linearize Eq. (2) about their steady-state values^44^. The Routh-Hurwitz criterion is used to determine the parameter value regions in which the steady-state solutions are stable. After some calculations we find that the fixed point of Eq. (4) is stable under the condition:


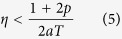


and the fixed point of Eq. (3) is stable for:


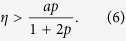


**Figure 1:**
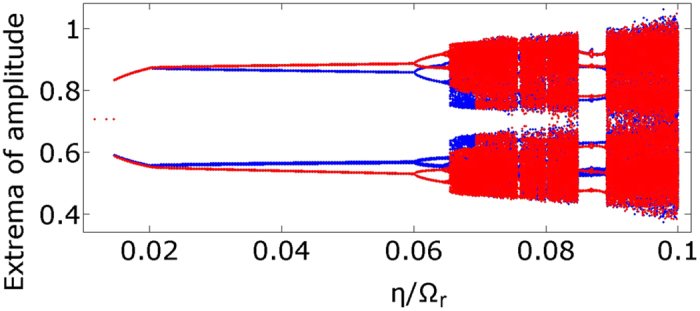
This figure depicts the amplitude maxima and minima of the electric field of two coupled diode lasers in dependence of the coupling strength *η* which has been rescaled to the relaxation oscillation frequency Ω_*r*_. The blue color refers to the first laser and the red color to the second one. The steady state, otherwise known as continuous wave operation, undergoes a Hopf bifurcation at 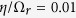 and, as a result, a limit cycle is born, that oscillates at the free running relaxation frequency. At 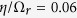, the system undergoes a period doubling bifurcation leading to a chaotic region which is interrupted by windows of periodic operation. Other parameters are: 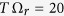, *p* = 0.5, 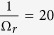, and *a* = 5.

In order to understand the effect of the coupling strength, Fig. 1 depicts a numerically obtained bifurcation diagram of the maxima and minima of the amplitude of the oscillating electric field. A Hopf bifurcation occurs at 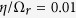 where the coupling strength is normalized to the relaxation oscillation frequency 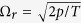
45. As the coupling is increased the limit cycle exists until 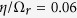. After that, a period-doubling cascade takes place, leading to chaos. The system remains chaotic until the approximate value of 0.084 and then enters a new limit cycle which is stable up to 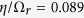, which is followed by a new period doubling cascade into a second chaotic region.

**Figure 2:**
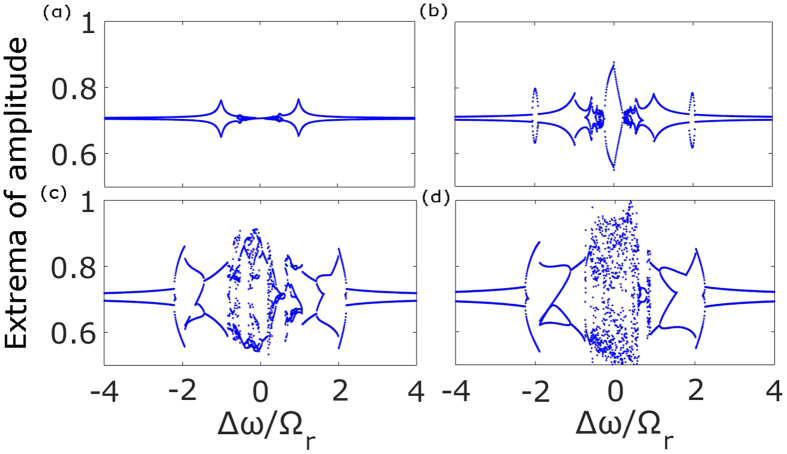
Extrema of the amplitude of the electric field in dependence of the detuning, for different values of the coupling strength. (**a**) 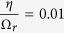, (**b**) 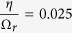, (**c**) 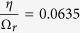, (**d**) 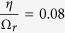. The difference in the detuning Δ*ω* has been rescaled by the relaxation oscillation frequency Ω_*r*_. In (**a**) we notice that the amplitude resonates at 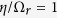, as expected. Such behavior shows the primary resonance of the system when the parametric driving frequency is equal to the internal frequency of the oscillator, i. e. the free running relaxation oscillation. In (**b**) we notice subharmonic resonances at 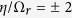, hysteretic behavior at the primary resonance, and at (**c**,**d**) the emergence of chaos is evident. Other parameters: 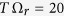, *p* = 0.5, 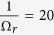 and *a* = 5.

Apart from the coupling strength, another crucial parameter is the optical frequency detuning and its correlation with the amplitude instability and mutual coherence of the light emitted by the laser. For both solid state^46^,^47^ and semiconductor lasers^40^, the complexity of the system increases immensely by introducing detuning. As expected, the most relevant parameter is actually the *difference* between the laser detunings rather than their individual values. The bifurcation diagram of Fig. 2 shows the maxima and minima of the electric field amplitude in dependence of 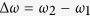, rescaled by the free relaxation frequency 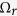. This has been repeated for various values of the coupling strength (Fig. 2(a–d)). We observe that in a certain range of 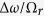 values the amplitude of the laser oscillations increases significantly. Moreover, for large coupling strengths (Fig. 2(c,d)) the behavior of the system is rich and complex in dynamical responses. It is also noticeable that although some *η* values render the system chaotic in the case without detuning (see Fig. 1), for the same coupling strengths the dynamics is regular in the presence of detuning (Fig. 2(d)).

The situation is much more complicated when we consider larger arrays. In the case of *M* coupled lasers, it can be found that the critical coupling strength, for the special case of the anti-phase region (see Eq. (6) for two coupled lasers), changes to^48^:


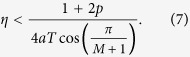


As *M* increases, the critical coupling decreases roughly as 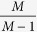 and reaches a limiting value at large *M* > 10 which is half of that corresponding to *M* = 2. Throughout this work, we will consider an array of 200 lasers. The numerical integration has been done by using the fourth order Runge-Kutta algorithm.

### Dynamics of coupled lasers with zero detuning

**Figure 3:**
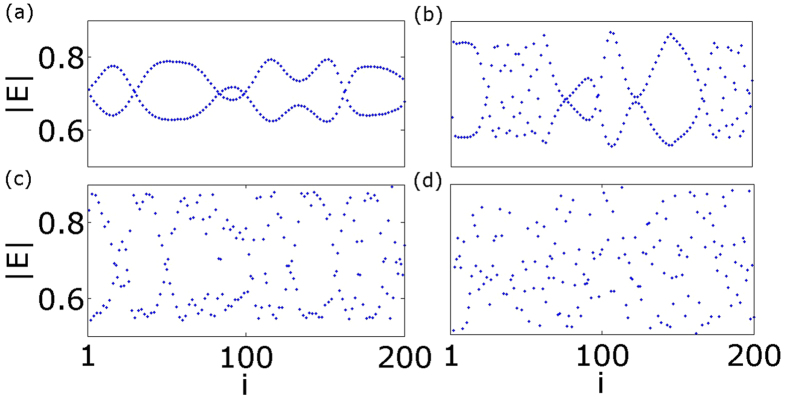
Snapshots of the amplitude of the electric field in an array of *M* = 200 lasers for different coupling strengths without detuning: (**a**) 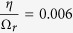, (**b**) 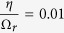, (**c**) 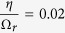, (**d**) 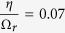. Other parameters: 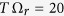, *p* = 0.5, 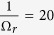 and *a* = 5. For low coupling strengths each laser is in anti-phase synchronization with its nearest neighbors (panel (a)). As the coupling increases the system enters the fully incoherent state (panel (d)).

First we focus on the influence of the coupling strength on the collective behavior, in the absence of detuning. We use the same initial conditions throughout the manuscript, namely random phases taken from a uniform distribution on the interval [−*π* to *π*], and fixed amplitudes 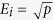 and inverse populations *N*_*i*_ = 0. According to Eq. (7), the Hopf bifurcation for our laser array occurs at the value 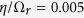. Slightly above this value, the system demonstrates a self-organized pattern (see Fig. 3(a,b)): The laser array splits into two sub-systems with each laser having a phase difference equal to *π* with its nearest neighbors (anti-phase synchronization^40^). This pattern gradually vanishes with increasing coupling strength and the system becomes fully incoherent (Fig. 3(c,d)). In Fig. 3 snapshots of the amplitude of the electric field are shown at 100 *T*_*r*_, where 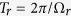 is the period of the relaxation oscillation of the free running diode laser.

### Effect of optical frequency detuning and chimera states

**Figure 4:**
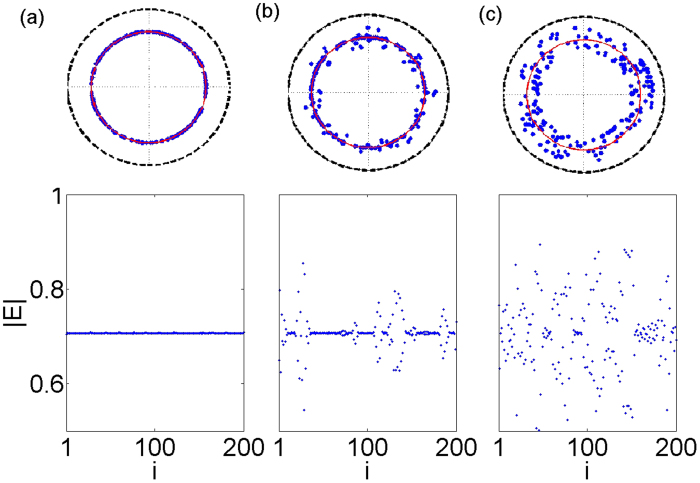
Top: The electric field in the complex unit circle for different coupling strengths and constant detuning. The red circle denotes the steady state solution where the amplitude of the oscillations is constant. Bottom: Corresponding snapshots of the amplitude of the electric field. (**a**) *H* = 0.008 (fully synchronized state), (**b**) *H* = 0.014, (amplitude chimera state), and (**c**) *H* = 0.026 (incoherent state). Other parameters: Δ = 0.01, 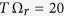, *p* = 0.5, 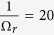 and *a* = 5. For further visualization refer to the Supplementary Movies S1,S2,S3.

The situation becomes significantly different when we consider finite optical frequency detuning. We incorporate detuning in the following way:


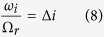


where Δ is a constant. With this distribution, the differences of the detuning have a simple form: 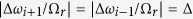
27. It is possible to realize different forms of synchronization depending on the coupling strength, which we redefine as 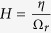. One case is full synchronization, where *E*_*i*_ = *E*_*j*_ holds for all lasers 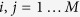 (see Fig. 4(a), bottom). The behavior is therefore similar to that of the uncoupled system since the whole array ends up in the steady state (each laser is lasing with constant intensity equal to 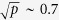). In a partially synchronized state the amplitudes are different in one or more lasers (see Fig. 4(b), bottom) and in the unsynchronized state there is no fixed amplitude relation between the oscillators (see Fig. 4(c), bottom). In Fig. 4(a–c) (top) we can see all of these states depicted in the complex unit circle. The red circle denotes the steady state solution where the amplitude of the oscillations is constant. In the top panel of Fig. 4(a) the amplitudes are locked to this value, while the phases of the individual lasers are randomly distributed over the steady state solution circle. This case corresponds to amplitude (intensity) synchronization. The opposite situation is full asynchrony, displayed in the top panel of Fig. 4(c), where both amplitude and phase exhibit incoherent behavior. The intermediate case is shown in Fig. 4(b) where an amplitude-chimera^49^ is illustrated through the coexistence of partial amplitude locking and incoherence. (For more information see Supplementary Movies S1,S2,S3 corresponding to Fig. 4(a–c)).

**Figure 5:**
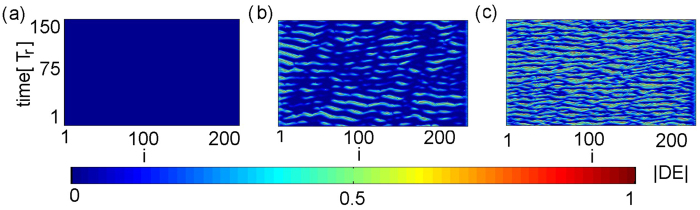
Spatio-temporal evolution of the local curvature *DE*_*i*_ (Eq. (9)) for different values of the coupling strength: (**a**) *H* = 0.008, (**b**) *H* = 0.014, (**c**) *H* = 0.026. Blue and red color denote full synchronization (*DE*_*i*_ = 0) and full incoherence (*DE*_*i*_ = 1), respectively. The spatio-temporal representation of the turbulent chimera state is shown in the middle panel. Other parameters as in Fig. 4.

In order to quantify the spatial coherence of the observed patters we calculate the local curvature *DE*_*i*_ (Eq. (9)). Figure 5 shows the spatio-temporal evolution of the local curvature corresponding to the states of Fig. 4. In the fully synchronized case the local curvature is equal to zero (Fig. 5(a)). In Fig. 5(b) we have the case of an amplitude-chimera state. We see that this is not a stationary pattern since the local curvature oscillates in time. The fully incoherent states is shown in Fig. 5(c), where the local curvature attains higher values.

**Figure 6:**
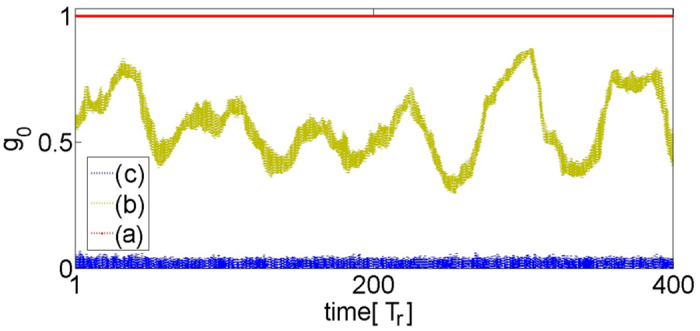
The time evolution *g*_0_(*t*) (Eq. 10) of the spatial extent occupied by the coherent lasers, for Fig. 5(a–c). In the fully synchronized state *g*_0_(*t*) is constant and equal to unity (**a**). The irregular oscillatory *g*_0_(*t*) is a signature for a turbulent chimera state (**b**). The incoherent state corresponds to *g*_0_(*t*) close to zero (**c**). Other parameters as in Fig. 4.

In Fig. 6, the time evolution of the spatial extent occupied by the coherent lasers, *g*_0_(*t*) (Eq. 10), for all three cases of Fig. 5 is plotted. We see that for the case of Fig. 5(b) *g*_0_ oscillates in an irregular manner, and therefore the corresponding amplitude chimera states are *turbulent* according to the classification scheme in ref. 5. The other two curves (a) and (c) refer to full synchronization and full incoherence, respectively.

**Figure 7:**
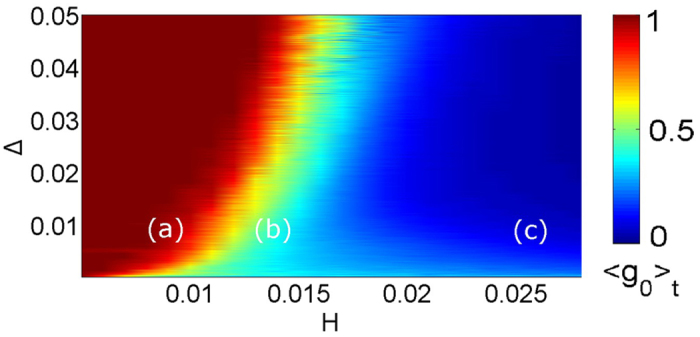
Dependence of the temporal mean 〈*g*_0_〉_*t*_ on parameters *H* and Δ. Points (**a**) (*H* = 0.008, Δ = 0.01), (**b**) (*H* = 0.014, Δ = 0.01), and (**c**) (*H* = 0.026, Δ = 0.01), correspond to Fig. 5(a–c). The boundary between full synchronization (red) and full desynchronization (blue) marks the regions where turbulent chimeras emerge. Other parameters as in Fig. 4.

**Figure 8:**
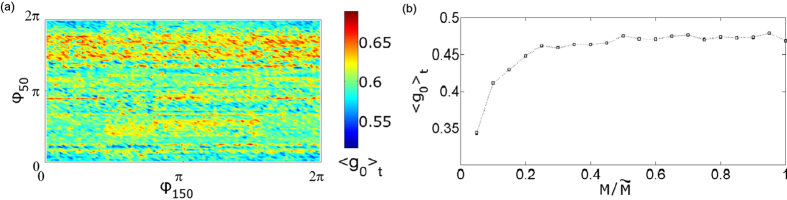
(**a**) The temporal mean 〈*g*_0_〉_*t*_ on the (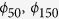)-projection. The initial conditions of the phases for all lasers are random and fixed except for 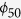 and 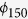 which are varied. (**b**) The temporal mean 〈*g*_0_〉_*t*_ as a function of the system size normalized to 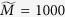. Parameters: *H* = 0.014, Δ = 0.01, 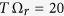, *p* = 0.5, 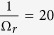 and *a* = 5.

The coaction of the detuning and the coupling strength on the observed synchronization patterns will be discussed next. In Fig. 7 the temporal mean of *g*_0_(*t*) (averaged over 400 *T*_*r*_) is plotted in the (*H*, Δ) parameter space. The initial conditions of the phases are randomly distributed between −*π* and *π*, while for the electric field amplitudes and the population inversions they are chosen identical for all lasers: 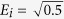, *N*_*i*_ = 0. The labels (a–c) mark the coordinates corresponding to Fig. 5(a–c), respectively. It is clear, that the parameter space is separated in two main domains, one of 〈*g*_0_〉_*t*_ values close to unity which corresponds to full coherence and contains point (a), and one of 〈*g*_0_〉_*t*_ values tending to zero which corresponds to full incoherence and contains point (c). On the boundary between these two areas, lies a small region where the amplitude chimeras arise. Note that, due to multistability, the mapping of the dynamical patterns may slightly change with different choice of initial conditions. The qualitative result, however, will be the same. For example, in Fig. 8(a), we plot 〈*g*_0_〉_*t*_ for a system with all initial phases randomly distributed but fixed, except those of laser 50 and 150, which we vary from 0 to 2*π*. Clearly, the exact values of 〈*g*_0_〉_*t*_ change but remain within the range allowing for chimera states.

Finally, the question of system size is addressed. In our simulations we observe that the behavior of the system does not change significantly when increasing *M* from 200 to 1000. This is illustrated in Fig. 8(b). After *M* > 200 the temporal mean 〈*g*_0_〉_*t*_ remains constant in time. From this fact we can conclude that, for an appropriately large system, the formation of chimera states is size-independent.

## Discussion

In conclusion, we have found amplitude chimera states in a large one-dimensional network of semiconductor lasers by properly modifying the optical frequency detuning. Local coupling is sufficient to generate these states. By using suitable classification measures we have quantified the observed dynamics. Due to the system’s multistability, even a slight change in the initial conditions may produce different values for these measures. However, the range of the obtained values ensures the existence of chimeras, the nature of which is turbulent. The system size also has an effect on the calculated values, which saturate for arrays with more that 200 emitters. A systematic study in the optical frequency detuning and coupling strength parameter space, shows that the region of chimera states lies between full synchronization and desynchronization. The ability to control the dynamics in and out of the synchronized state, may have multiple technological applications regarding the generation of on demand diverse waveforms^50^. For future studies, it would be worthwhile to explore this, as well as the effects introduced by noise and the laser pump power, which is the most conveniently accessible control parameter in chip scale diode systems.

## Methods

Recently, Kemeth *et al*. presented a classification scheme for chimera states^5^. For measuring spatial coherence, in particular, they introduced a quantity called *local curvature* which may be calculated at each time instance. This is done by applying the discrete Laplacian *DE* on the spatial data of the amplitude of the electric field:


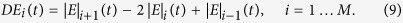


In the synchronization regime the local curvature is close to zero while in the asynchronous regime it is finite and fluctuating. Therefore, if *g* is the normalized probability density function of |*DE*|, *g*(|*DE*| = 0) measures the relative size of spatially coherent regions in each temporal realization. For a fully synchronized system *g*(|*DE*| = 0) = 1, while for a totally incoherent system it holds that *g*(|*DE*| = 0) = 0. A value between 0 and 1 of *g*(|*DE*| = 0) indicates coexistence of synchronous and asynchronous lasers.

The quantity *g* is time-dependent. Complementary to the local curvature we also calculate the spatial extent occupied by the coherent lasers which is given by the following integral:


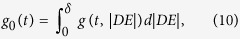


where *δ* is a threshold value distinguishing between coherence and incoherence which is related to the maximum curvature and is system-dependent. We will apply these measures in order to classify the observed patterns and we will discuss their dependence on the coupling strength *H* and the detuning parameter Δ.

## Additional Information

**How to cite this article:** Shena, J. *et al*. Turbulent chimeras in large semiconductor laser arrays. *Sci. Rep.* **7**, 42116; doi: 10.1038/srep42116 (2017).

**Publisher's note:** Springer Nature remains neutral with regard to jurisdictional claims in published maps and institutional affiliations.

## Supplementary Material

Supplementary Information

Supplementary Movie S1

Supplementary Movie S2

Supplementary Movie S3
