# Supplementary material for: Turbulent chimeras in large semiconductor laser arrays
Source: Sci Rep. 2017 Feb 6;7:42116. doi: 10.1038/srep42116 (PMC5292712; doi:10.1038/srep42116)
Supplement: Supplementary Information [file srep42116-s1.pdf]

Turbulent chimeras in large semiconductor laser arrays

J. Shena[1], Hizanidis[1,*], V. Kovanis[2], G. P. Tsironis[1,3,4]

[1]Crete Center for Quantum Complexity and Nanotechnology, Department of Physics, University of Crete, 71003 Heraklion, Greece
[2]Department of Physics, School of Science and Technology, Nazarbayev University, 53 Kabanbay Batyr Ave, Astana, Republic of Kazakhstan
[3]Institute of Electronic Structure and Laser, Foundation for Research and Technology–Hellas, P.O. Box 1527, 71110 Heraklion, Greece.
[4]National University of Science and Technology MISiS, Leninsky prosp. 4, Moscow, 119049, Russia
*hizanidis@physics.uoc.gr

Supplementary Information

Supplementary Movies

We provide here the details of the three Supplementary Movies which correspond to Fig. 4((a)-(c)) in the main text. They illustrate the dynamical evolution of synchronous (Fig. 4(a)), turbulent chimera (Fig. 4(b)) and unsynchronous (Fig. 4(c)) dynamics.

Supplementary Movie S1 (File movie S1.avi)

It shows the time evolution of the coupled lasers model, as it is described by Eqs. (1) in the main text. The electric field in the complex unit circle (left) and the amplitude of the corresponding oscillations (right) is shown, for coupling strength $H = 0.008$. The dynamical behavior shown in this movie is synchronous (see also Fig. 4(a) in the main text). It is similar to that of the uncoupled system since the whole array ends up in the steady state.

Supplementary Movie S2 (File movie S2.avi)

It shows the time evolution of the coupled lasers model, as it is described by Eqs. (1) in the main text. The electric field in the complex unit circle (left) and the amplitude of the corresponding oscillations (right) is shown, for coupling strength $H = 0.014$. The dynamical behavior shown in this movie corresponds to a turbulent chimera state (see also Fig. 4(b) in the main text).

Supplementary Movie S3 (File movie S3.avi)

It shows the time evolution of the coupled lasers model, as it is described by Eqs. (1) in the main text. The electric field in the complex unit circle (left) and the amplitude of the corresponding oscillations (right) is shown, for coupling strength $H = 0.026$. The dynamical behavior shown in this movie is asynchronous (see also Fig. 4(c) in the main text).
